# Human brucellosis: An overview

**Published:** 2015

**Authors:** Mohammad Reza Hasanjani Roushan, Soheil Ebrahimpour

**Affiliations:** 1 Infectious Diseases and Tropical Medicine Research Center, Babol University of Medical Sciences, Babol, Iran

Brucellosis is a common zoonotic infection caused by bacterial genus brucella. Brucellosis is an old disease known by various names including undulant fever or Mediterranean fever. This is one of the infectious diseases transmissible between animals and humans. Brucella bacteria multiply inside the body in some ways like swallowing, breathing, contact between damaged skin and slinked fetus or amniotic fluid of septic animals (1, 2). This infection is more common in Mediterranean areas, the south and the center of America, Africa, Asia, Arab peninsula, Indian subcontinent and the Middle East. The annual incidence rates per million population. In some endemic regions, the rate of brucellosis are as follows: Saudi Arabia (214.4), Iran (238.6), Turkey (262.2), Iraq (278.4), and certainly the maximum incidence in the world had been reported in Syria (1603.4) (3), But according to World Health Organization (WHO) the real incidence is 10-25 times more than what have been reported (4, 5). Processing milk, climate condition, hygienic environment, economic and social conditions are the most effective factors in the infection and transmission of brucellosis. Human contact with infected domestic animals is often a transmission route of Brucellosis infection. The incubation period of this infection is between 1-3 weeks but can take several months before clinical disease appearance. The most common nonspecific symptoms of brucellosis are fever, night sweats, asthenia, insomnia, anorexia and headache. For detecting patients suffering from brucellosis, there medical history should be reviewed, therefore these kinds of tests can be proposed such as: routine biochemical, hematology tests, echo cardiography, brucella cultures, serological, molecular tests and other modalities. It is necessary to mention that bone marrow (BM) culture in some studies is the current gold standard method for confirming a case of brucellosis. In the acute form of brucellosis, the sensitivity of brucella blood cultures has been reported as 80%-90%. Whereas, in the chronic form, sensitivity has been introduced as 30%-70% (6). Rose Bengal test is so fast, but this test has many false-negative results in its chronic form (7). Serum agglutination test (SAT) is the most common acceptable serological diagnostic test for human brucellosis. Undoubtedly, in endemic areas the use of SAT titer ≥ 1:320 and titer 2- mercoptoetanol (2ME) ≥ 1:160 are more appropriate. It is necessary to explain that definitive treatment of patients has a correlation in declining SAT titers (8). Coombs test is useful for the diagnosis of cases with relapses. Lateral flow assay is used to overlook the patient in endemic regions and this test provides fast results (7, 9). Molecular tests like polymerase chain reaction (PCR) has generally spread for the diagnosis of brucella some decades ago. Today, this test is mostly used for the evaluation of treatment efficacy. WHO in 1986, for acute brucellosis treatment in adults recommended therapeutic regimen including: rifampicin 600-900 mg plus doxycycline 100 mg for 6 weeks (10). Some studies showed that the combination of edible doxycycline for 45 days with intramuscular gentamicin for 7 days have the same influence as doxycycline for 45 days with streptomycin for 14 days (11). Some experts on patients with brucellous under the age of 60 with peripheral arthritis, sacroilitis and epidydimoorchitis symptoms suggested streptomycin with doxycycline or the combination of gentamicin and doxycycline for the patients over the age of 60 likewise, those the same symptoms mentioned with the combination of refampin and doxycycline (12). Furthermore, these organisms are resistant to the above medications and should be determined.
